# Artery-First Approach During Minimally Invasive Pancreatoduodenectomy for Pancreatic Cancer: Outcomes from a Single Center and Comparison Between Laparoscopic and Robotic Approaches

**DOI:** 10.3390/cancers17132103

**Published:** 2025-06-23

**Authors:** Michele Mazzola, Michele Paterno, Alessandro Giani, Pietro Calcagno, Andrea Zironda, Gaia Mucci, Camillo Franzetti, Paolo De Martini, Giovanni Ferrari

**Affiliations:** ASST Grande Ospedale Metropolitano Niguarda, Division of Minimally-Invasive Surgical Oncology, Piazza Ospedale Maggiore, 3, 20162 Milan, Italy; michele.paterno2@ospedaleniguarda.it (M.P.); alessandro.giani@ospedaleniguarda.it (A.G.); pietro.calcagno@ospedaleniguarda.it (P.C.); andrea.zironda@ospedaleniguarda.it (A.Z.); gaia.mucci@ospedaleniguarda.it (G.M.); camillo.franzetti@ospedaleniguarda.it (C.F.); paolo.demartini@ospedaleniguarda.it (P.D.M.); giovanni.ferrari@ospedaleniguarda.it (G.F.)

**Keywords:** laparoscopic pancreatoduodenectomy, robotic pancreatoduodenectomy, minimally invasive pancreatoduodenectomy, pancreatic surgery, artery-first approach, pancreatic cancer

## Abstract

**Background:** Despite benefits during open pancreatoduodenectomy (PD), the artery-first approach (AFA) during minimally invasive PD (MIPD) has been poorly investigated. **Methods:** Data of consecutive patients undergoing MIPD (both laparoscopic (LPD) and robotic (RPD)) from 2020 to 2024 for pancreatic cancer (PC) were prospectively collected and retrospectively analyzed, comparing the surgical and oncological outcomes of LPD with right AFA and RPD with posterior AFA. The rate of ineffective AFA (IAFA), defined in the case of excessive resection time, estimated blood loss, or conversion to laparotomy, was also investigated. **Results:** 71 patients undergoing MIPD were selected (32 LPD and 39 RPD). Baseline patients’ characteristics only differed for a higher rate of neoadjuvant treatment in LPD and RPD groups, respectively (23.1% vs. 0%, *p* = 0.0036). No patients underwent conversion. R0 resection was obtained in 74.6% of patients. No difference between the groups was found regarding intraoperative, postoperative, and oncological outcomes except for a greater number of lymph nodes harvested in RPD (24 vs. 17, *p* = 0.023). IAFA was observed in 12.7% of patients, without difference between the groups (9.4 vs. 15.4%, *p* = 0.499, in LPD and RPD, respectively). **Conclusions:** MIPD with AFA was feasible and safe in patients affected by PC. RPD using posterior AFA had a higher number of lymph nodes retrieved when compared to LPD using right AFA. RPD has provided subjective advantages for the surgeon in terms of handling, safety, and reproducibility, although these have not translated into better outcomes.

## Simple Summary

Pancreatic cancer remains one of the most challenging periampullary malignancies to treat due to its late-stage diagnosis and complex surgical resectability. The Artery-First Approach (AFA) is a promising strategy in pancreatic surgery; however, its feasibility and reproducibility during Minimally Invasive Pancreatoduodenectomy (MIPD) remain under investigation. The present study analyzed 71 patients undergoing AFA during both laparoscopic and robotic pancreatoduodenectomy (LPD and RPD). It showed that both right and posterior AFA are feasible during MIPD for pancreatic cancer and reported a comparison of LPD and RPD outcomes. Although no significant differences in surgical and oncological outcomes were observed, RPD had enormous advantages in terms of enhanced surgical ergonomics, precision, and ease of dissection in tight operative fields, contributing to its adoption as the preferred approach for MIPD. This study may provide valuable insights into the role of AFA when performed using minimally invasive surgery.

## 1. Introduction

Pancreatoduodenectomy (PD) is the mainstay of treatment for periampullary tumors [1,2]. The superior mesenteric artery (SMA) represents a crucial vascular landmark during PD for several reasons. The neoplastic involvement of the SMA is one of the main causes of the local unresectability of periampullary neoplasms [3]. Moreover, the SMA is considered the medial boundary of the mesopancreas and its lateral aspect should be dissected in order to guarantee an adequate oncological radicality [4,5]. Furthermore, the SMA is deeply located, dorsally to the superior mesenteric vein (SMV), and surrounded by frail vessels, making its dissection challenging and at high risk of intraoperative bleeding [4,6,7,8]. The role of the SMA as a vascular landmark during PD has progressively gained attention, offering the rationale for a novel surgical strategy, namely the artery-first approach (AFA) [9]. It is based on both the early identification of the SMA and the early ligation of the peripancreatic vessels, especially the inferior pancreatico-duodenal artery (IPDA), allowing a correct resectability assessment prior to any irreversible procedure, and an in-flow control [1,10]. Six main types of AFA have been described for open PD, each of them showing particular advantages in specific conditions according to the tumor site, patient’s anatomical features, and surgeon’s preference [11]. Minimally invasive approaches for pancreatic resections, including minimally invasive PD (MIPD), have been progressively adopted in the treatment of periampullary tumors with encouraging results; however, few reports focused on the approach to the SMA during MIPD [12,13]. Pancreatic cancer (PC) is the most frequent type of periampullary tumor [14]. Due to its particular behavior, consisting of a more advanced stage at diagnosis and a tendency to infiltrate the nerve sheaths, PC usually has a worse prognosis, a lower resectability rate, and requires a more demanding surgery, making it the perfect scope for AFA [15]. The aim of this study was to investigate the outcomes of AFA in patients undergoing MIPD for PC, in a Western tertiary referral center. Preoperative factors associated with AFA efficacy were also investigated.

## 2. Materials and Methods

### 2.1. Study Overview

Since September 2020, laparoscopic PD (LPD) with AFA has been offered to all patients who are candidates for PD in our division, with the exemption of those presenting with major vascular involvement on preoperative imaging or anesthesiologic contraindications to pneumoperitoneum. The robotic approach was introduced in our practice in September 2022, after an adequate learning curve in other fields of surgery (colorectal, oesophagogastric, and left pancreatic resections), and has rapidly become the only minimally invasive approach for PD. Selection criteria for MIPD gradually evolve over time also including patients undergoing neoadjuvant treatment and with limited vascular involvement. Data of consecutive patients who underwent MIPD (both laparoscopic and robotic) from September 2020 to March 2024 for PC were prospectively collected and retrospectively analyzed. The study protocol followed the ethical guidelines of the 1975 Declaration of Helsinki (as revised in Brazil in 2013). Results are reported according to Strengthening the Reporting of Observational Studies in Epidemiology (STROBE) [16].

### 2.2. Surgical Technique and Perioperative Management

#### 2.2.1. LPD

**Figure 1:**
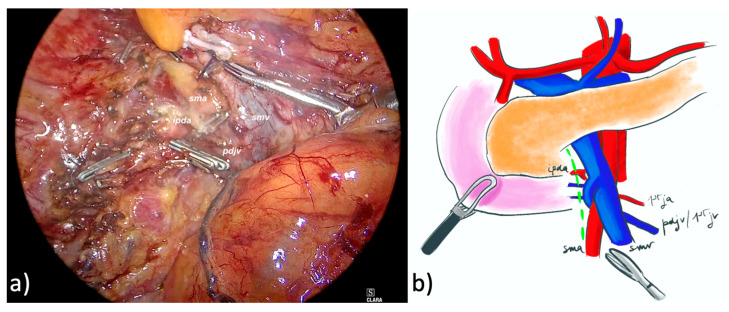
Dissection of the mesopancreas using laparoscopic lateral artery-first approach. (**a**) intraoperative vision of the surgical field with right lateral aspect of the superior mesenteric vein (smv) and the superior mesenteric artery (sma). (**b**) Schematic illustration showing smv, sma, inferior pancreatico-duodenal artery (ipda), first jejunal artery (1st ja), proximal dorsal jejunal vein (pdjv), first jejunal vein (1st jv).

The whole description of the technique adopted for LPD was previously reported and is here briefly described [17,18]. A modified right AFA using the proximal dorsal jejunal vein preisolation method was used [19]; it was based on the early sealing of the right gastroepiploic vein and the wide dissection of the SMV on its right lateral aspect until the proximal dorsal jejunal vein was identified and its pancreatic tributaries divided (Figure 1).

The SMA was identified behind the proximal dorsal jejunal vein and dissected along its right aspect through its origin. The lymphovascular tissue between the uncinate process and the anterior-right lateral aspect of the SMA was removed; the IPDA was identified and sealed with clips. After an extensive Kocher maneuver, the Treitz ligament was divided and the first jejunal loop was de-rotated beneath the mesenteric axis and sectioned. The gastric antrum or duodenum and the gastroduodenal artery were sectioned using a stapler. The pancreatic neck was underpassed and divided using cold scissors, as well as the common hepatic duct. Frozen sections of both pancreatic and bile duct stumps were always performed to confirm negative resection margins. After ligation of the superior pancreatico-duodenal veins, the SMV was shifted to the patient’s left by the first assistant, and all the tissue between the celiac trunk and the SMA was removed completing the mesopancreas excision. The fistula risk score (FRS) was calculated [20]. Pancreaticojejunostomy was performed according to Cattell-Warren or Blumgart, depending on the surgeon’s preference and regardless of the surgical approach [21]. A single-layer end-to-side hepaticojejunostomy was performed. After antecolic transposition, manual double-layer hand-sewn duodenojejunostomy or mechanic gastrojejunostomy was performed. One or two spiral drains entering the right side of the patient were positioned near the pancreaticojejunostomy. At the end of the procedure, the nasogastric tube was removed. An intraoperative near-zero fluid balance was applied. According to institutional protocol, patients’ mobilization and liquid diet were allowed when tolerated. On postoperative day 2 patients received a solid diet. Drains were mobilized and removed according to an early-removal policy in case of negative drain fluid amylase and reassurance fluid characteristics [22].

#### 2.2.2. Robotic PD (RPD)

**Figure 2:**
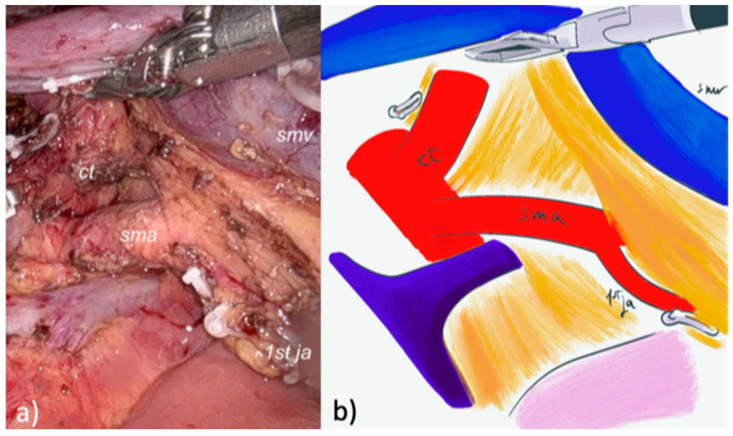
Dissection of the mesopancreas using robotic posterior artery-first approach. (**a**) intraoperative vision of the surgical field with the posterior aspect of the superior mesenteric vein and right lateral aspect of the superior mesenteric artery. (**b**) Schematic illustration showing superior mesenteric vein (smv), superior mesenteric artery (sma), celiac trunk (ct), first jejunal artery (1st ja).

**Figure 3:**
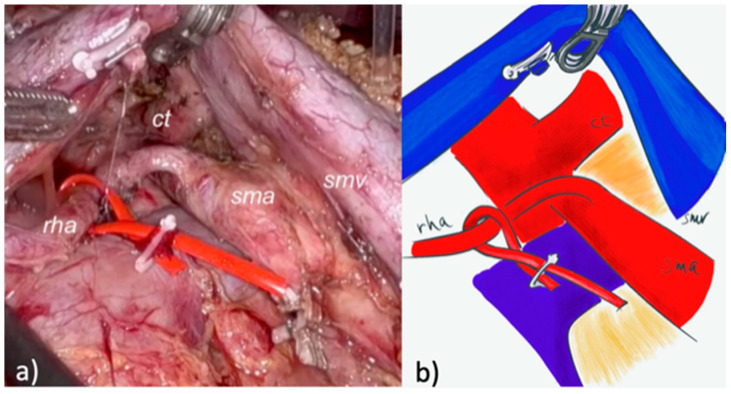
Completion of mesopancreas excision using robotic posterior artery-first approach, in patient with aberrant hepatic artery from superior mesenteric artery. (**a**) intraoperative vision of the surgical field with superior mesenteric vein/portal vein (blu vessel loops) and the aberrant hepatic artery (red vessel loop). (**b**) Schematic illustration showing superior mesenteric vein (smv), superior mesenteric artery (sma), replaced right hepatic artery (rha).

A pure robotic approach (4 robotic trocars and 2 laparoscopic trocars) with the Da Vinci™ Xi system (Intuitive Surgical^®^, Inc., Sunnyvale, CA, USA) was used for RPD. A modified posterior AFA was used. After the gastrocolic ligament was opened and the right colonic flexure mobilized, an extensive Kocher maneuver was carried out exposing the inferior vena cava and the left renal vein. The right crura was exposed and the right aspect of the celiac trunk was identified and cleared. The SMA was identified at its origin and dissected in a cranial-to-caudal fashion, following a periadventitial plane, until the IPDA was identified at its origin and sealed with clips (Figure 2 and Figure 3).

The first jejunal loop was then de-rotated through the Treitz window and sectioned using a stapler. The tributary veins from the uncinate process to the proximal dorsal jejunal vein, or directly to the SMV, were transected, and the dissection of the posterior mesopancreas was completed exposing the dorsal aspect of the SMV (Figure 2). After the gastric antrum or duodenum and the gastroduodenal artery were sectioned using a stapler, a complete lymphadenectomy of stations 8–9–12 was performed. The pancreatic neck was underpassed and divided using cold scissors, as well as the common hepatic duct. After ligation of the right gastroepiploic vein, the remaining pancreatico-duodenal veins were sectioned with the removal of all the tissue between the celiac trunk and the SMA, completing the mesopancreas excision. The following steps were the same as those for LPD.

### 2.3. Definitions and Variables

Patients’ baseline characteristics also included surgical approach (laparoscopic or robotic) and the presence of anatomical vascular variations in the hepatic artery at the preoperatively contrast-enhanced computed tomography imaging. Types III, IV, VI, VII, VIII, and IX of anomalies according to Michels’ classification were considered as aberrant right hepatic arteries. Surgical outcomes were associated with procedures, vascular resection, conversion rate, duration of resection phase, and overall operative time. Conversion from MIPD to open surgery was defined as the need to complete the resection phase by any type of laparotomy. The FRS was calculated based on pancreatic texture measured by palpation of the extracted specimen, Wirsung diameter assessed intraoperatively at the time of transaction, estimated blood loss, and pathology [20]. The pathological radicality of resection, pTNM, and pathologic stage were classified according to the 8th edition of the American Joint Committee on Cancer staging system [23]. A minimum of 15 lymph nodes (LNs) harvested was chosen to consider the lymphadenectomy adequate [24]. Resection margins were defined according to the Royal College of Pathologists; margin status was considered R1 when the distance between the tumor and any resection margins was ≤1 mm. The Clavien-Dindo classification was used to grade postoperative complications; those graded > 2 were considered severe [25]. Pancreas-specific complications were recorded separately and defined in accordance with the International Study Group of Pancreatic Surgery [26,27,28]. Ineffective AFA (IAFA) was defined in the case of resection time or estimated blood loss > 90th percentile, or conversion to laparotomy.

### 2.4. Study Endpoints

The primary endpoint was IAFA during MIPD. The secondary endpoints were surgical outcomes (conversion rate, duration of resection phase and overall operative time, postoperative complications), pathological outcomes (radicality of resection, number of LNs harvested), and identification of factors associated with IAFA in patients undergoing MIPD. A subanalysis comparing patients undergoing LPD and RPD was also performed.

### 2.5. Statistical Analysis

Data are expressed as median and interquartile range and number and relative percentage. The normal distribution of continuous variables was assessed with the Shapiro–Wilk test. Univariate analysis was carried out to identify variables which were independently associated with IAFA; the variables with *p* values < 0.10 at univariate analysis were further analyzed with multivariable logistic regression. Categorical variables were analyzed using the Fisher exact test or Chi-Square test, as appropriate, while continuous ones were analyzed using the Student t test or Mann–Whitney test. *p* Values were considered significant when <0.05. Data analysis was performed using JMP^®^, Version 16.0 (SAS Institute Inc., Cary, NC, USA, 1989–2019).

## 3. Results

### 3.1. Patient Selection

In the study period, 209 patients underwent PD and 109 of these were treated for PC. After the exception of 38 patients receiving open PD, 71 patients undergoing MIPD were selected: 32 underwent LPD and 39 RPD. Baseline patients’ characteristics are shown in Table 1.

No difference was found between the subgroups (LPD and RPD) in terms of baseline characteristics (sex, age, BMI, ASA score, Age-adjusted Charlson Comorbidity Index, previous abdominal surgery, major venous involvement, vascular anomalies, and preoperative biliary drainage). More patients in the RPD group received neoadjuvant treatment (23.1% vs. 0%, *p* 0.0036).

### 3.2. Operative and Postoperative Outcomes

Intraoperative outcomes are shown in Table 2.

No patients underwent conversion. The median operative time and resection time of the entire cohort were 545 min and 310 min, respectively. The median estimated blood loss was 250 mL. The majority of patients had an intermediate or low risk of fistula based on FRS. No difference between the groups was found regarding intraoperative outcomes. Postoperative outcomes are reported in Table 3.

The overall and severe complication rate of the entire cohort was 45.1% and 21.1%, respectively. Four patients (5.6%) required reoperation. Pancreatic fistula and postpancreatectomy hemorrhage occurred in 8.4% and 9.9% of patients, respectively. The median length of stay was 11 days with an 8.4% readmission rate. The groups did not differ in terms of postoperative outcomes. IAFA was observed in 12.7% of patients, without difference between the groups (9.4 vs. 15.4%, *p* 0.499, in LPD and RPD, respectively). The main causes of IAFA were excessive blood loss (2 and 3 patients in LPD and RPD, *p* 1.000, respectively) and resection time (1 and 3 patients in LPD and RPD, *p* 0.622, respectively) without difference between the groups. At univariate analysis, male sex and ASA > 2 were detected as possible factors associated with IAFA (*p* < 0.10) and were included in the logistic regression. In multivariable analysis, only male sex was an independent predictor of IAFA. Univariate and multivariable analyses for IAFA are reported in Table 4.

### 3.3. Pathological and Oncological Outcomes

Pathological and oncological outcomes are reported in Table 5. R0 resection was obtained in 74.6% of patients. No R2 resection was reported. Among patients with R1 resection, posterior and medial resection margin was involved in 19.7% and 5.6%, respectively. The RPD group showed a greater number of LNs harvested (24 vs. 17, *p* = 0.023), with no difference between the groups regarding the median number of positive LNs (*p* = 0.630) and LNs ratio (*p* = 0.291). The rate of patients receiving adjuvant therapy was 59.7%, with no difference between patients undergoing LPD and RPD. No difference between the groups was found in terms of tumor features and stage.

## 4. Discussion

The present study showed that MIPD with AFA was feasible and safe for the treatment of patients affected by PC. Male sex was an independent predictor of IAFA. No difference was observed in patients undergoing MIPD regardless of the approach, except for a higher number of LNs harvested with RPD.

AFA has been proposed as a novel surgical strategy with potential advantages during PD, namely early determination of resectability and control of vascular in-flow for the pancreatic head, higher R0 rate, and avoiding vascular injury in patients with arterial anomalies [1,29]. Some doubts about the usefulness of AFA have been raised by a randomized study that showed no benefits in comparison to conventional PD [30]. However, a recent meta-analysis reported better outcomes for patients undergoing PD with AFA in comparison with standard PD regarding both overall and pancreas-specific complications, blood loss, length of stay, and margin radicality, suggesting potential clinical benefit for patients undergoing AFA in terms of postoperative recovery and oncological outcomes [1]. Considering that all the studies included in the meta-analysis selected exclusively patients undergoing open PD, the efficacy of AFA remains debated for MIPD [1]. Even though MIPD may ideally represent an optimal application for AFA, due to a careful dissection along embryological and periadventitial planes, the need to operate in very limited and tight spaces and the difficulty of performing effective tractions and counteractions could limit its feasibility, hindering the reproducibility of the results obtained during open PD. In the present series, AFA was always feasible during MIPD, with no conversion. The rate of IAFA was 12.7% and it was mainly related to excessively long resection time or blood loss; this data should be analyzed considering the characteristics of patients all affected by PC. In a previous study, patients affected by PC showed a trend through longer operative time and higher blood loss, even if not statistically relevant, as compared with those affected by other periampullary tumors [18]. However, these potential limits did not hinder patients’ safety as demonstrated by the absence of conversion, the adequacy of LN retrieval, and the acceptable postoperative outcomes in terms of complications and length of stay. The only factor associated with IAFA was male sex, while BMI and previous abdominal surgery were not. Many authors consider high BMI or previous abdominal surgery as exclusion criteria for MIPD. Interestingly, in the present study, neither of these variables was associated with IAFA; instead, male sex was a significant factor. This finding may suggest that, when performed by experienced surgeons, no absolute exclusion criteria should be applied to MIPD. However, caution should be applied when performing MIPD in males with high BMI, as these factors are associated with visceral fat and thickened mesentery [31]. Both these factors can make the procedure particularly challenging, potentially leading to longer operative time and increased blood loss. In particular, visceral obesity has proved to correlate with more complex procedures and increased postoperative complications after PD. Some authors even suggested considering the visceral fat area as a factor influencing surgical difficulty during robotic distal pancreatectomy [32]. Furthermore, the male sex has been recently proposed as a risk factor for postoperative pancreatic fistula in the setting of MIPD due to its association with visceral fat, fatty pancreas, and more challenging reconstructive phase [33].

A recent review identified a few studies focusing on AFA during MIPD, describing four main approaches to the SMA: anterior, posterior, right, and left [13]. Among these, the right AFA was the most frequently used due to the wider working space and the easy accessibility to the SMA; however, the right approach requires wide isolation of the anterolateral aspect of the SMV at an early phase of the dissection and the section of the tributary veins moving from the pancreatic head to the first jejunal vein or directly in the SMV, with potential risk of time-consuming and harmful bleeding, especially in obese patients with thickened mesenteric roots [19]. On the contrary, only a few studies reported experiences regarding the left approach, probably due to the difficulty in creating an adequate working space between the transverse mesocolon and the small intestine [34,35,36]. The anterior approach was described in a single case series, which underlined its usefulness in particular when facing major venous involvement needing resection [37].

The potential advantages of the posterior approach include the exploration and dissection of the SMA before venous evaluation, the possibility of performing an interaortocaval LNs biopsy, a safer probing of the SMV, and a more conducive lymphadenectomy of the hepatoduodenal ligament [38]. Accordingly, many authors advocated for the efficacy and potential benefits of the posterior approach during both open and MIPD, allowing for safer identification of the SMA at its origin, easy clearance of the lymphatic tissue on the right lateral aspect of the SMA and celiac trunk, and earlier assessment of disease extension and arterial control prior to sectioning the pancreatic neck or dissecting the SMV [11,39]. However, the dissection of the posterior plane along the anterior aspect of the aorta and above the left renal vein may not always be easy during open surgery, especially in the case of obese patients, due to the difficulty of moving the duodenopancreatic block on the left and the ventral-to-dorsal approach. During LPD, especially in patients with thickened retropancreatic tissues as in cases of pancreatitis, the posterior approach may even be unfeasible because of the rigid and straight instruments.

Only anecdotal reports focusing on AFA during RPD have been published in the literature. While previous studies have compared the surgical and oncological outcomes of patients undergoing open and minimally invasive resection for PC, no studies have specifically examined differences between the AFAs used for RPD and LPD [13,40,41]. Robotic surgery has shown clear advantages in comparison to laparoscopic one in terms of articulation of instruments, endowrist technology, mitigation of unintentional tremors, and immersive enhanced three-dimensional vision [42,43]. Some authors have argued that the advantages of robotic surgery could be more evident as the complexity of the intervention increases, suggesting an ideal field of application for pancreatic surgery [44]. Consistently, the implementation of robotic surgery was associated with improved outcomes during pancreatic and liver surgery; in particular, shifting from LPD to RPD resulted in a reduced operative time and blood loss and a higher number of retrieved LNs [45]. For adequate nodal staging, standard lymphadenectomy, with the removal of at least 15 LNs, is recommended because the LN status is an important prognostic factor, especially for patients affected by PC [45]. In the present study, even though an adequate number of LNs was harvested by using both approaches, RPD was associated with a greater number of LNs retrieved probably explained by a magnified visualization and a more precise dissection; however, no difference between the groups was found regarding LNs ratio. On the contrary, R0 was slightly in favor of LPD, even if not reach statistical significance; considering the number of LNs harvested, which confirmed a meticulous dissection, this data could be related to progressive modification of the inclusion criteria for MIPD, justified by the increasing acquisition of technical skills and the implementation of the robotic approach, as witnessed by the higher number of patients receiving neoadjuvant treatment and a trend in a greater number of vascular involvement and history of previous surgery in patients undergoing RPD. However, the R1 rate reported in the literature for patients affected by PC ranged between 30 and 35% in both open and MIPDs, which was consistent with our results (18.8% and 30.8% in LPD and RPD, respectively).

A report from a prospective national registry confirmed that the application of the robotic platform to pancreatic surgery was associated with reduced rates of conversion and blood loss and favored a wider diffusion of MIPD [46]. The present study well reflects the global trend in shifting from LPD to RPD; since its introduction in our clinical practice in 2022, RPD has rapidly become the only approach for MIPD. Although no significant difference was found in surgical outcomes between LPD and RPD, the benefit perceived by the surgeon in terms of safety, reproducibility, ease of instrument handling, and ergonomics has led to a complete abandonment of the laparoscopic approach in favor of the robotic one.

This study has several limitations that warrant caution in interpreting the results. The data were analyzed retrospectively and based on a single-center experience. The findings may have been influenced by changes in perioperative management and surgical expertise over time; in particular, the outcomes of RPD could have benefited from the learning curve previously established with LPD [21]. On the other hand, LPDs were performed by an experienced laparoscopic surgeon with more than 100 laparoscopic gastrectomies and 300 laparoscopic colectomies [21]. The same surgeon initiated the robotic program and began performing RPD after a structured training program, but with limited robotic experience. Therefore, the different learning curve for each approach, which was much higher for LPD, may have influenced the results in favor of LPD. A higher rate of neoadjuvant therapy was observed in the RPD group due to a gradual change in selection criteria over time. Although the influence of this specific factor on the study outcomes is impossible to analyze and beyond the aim of this study, no difference was observed between the groups in the resectability rate as demonstrated by the similar results in terms of blood loss, conversions, operative time, and R0. This could suggest, at most, a potential benefit of RPD in managing more complex conditions. Additionally, the data were derived from a single center with specialized expertise in advanced minimally invasive and pancreatic surgery, which may limit their generalizability to other settings. For each minimally invasive approach (laparoscopic and robotic), an AFA was used, restricting the ability to identify potential advantages of other approaches. The small sample size could introduce a risk of type II error.

## 5. Conclusions

This paper reported outcomes of MIPD with AFA showing its feasibility and safety in patients affected by PC. When comparing RPD with posterior AFA and LPD with right AFA no difference was found in terms of surgical and oncological outcomes, despite RPD having a higher number of LNs retrieved. RPD has provided subjective advantages for the surgeon in terms of handling, safety, and reproducibility, although these have not translated into better results with respect to the defined outcomes.

## Figures and Tables

**Table 1:** Baseline characteristics of patients undergoing MIPD.

Characteristic	Overall (71)	LPD (32)	RPD (39)	*p*
Sex (F)	39 (54.9)	19 (59.4)	20 (51.3)	0.495
Age (years) *	72 (65–80)	73 (60.5–80.7)	71 (65–79)	0.556
BMI (kg/m^2^) *	24.7 (22.5–27.6)	25.6 (22.6–27.8)	24.6 (22.0–26.7)	0.291
ACCI *	5 (4–6)	5 (4–6)	5 (4–6)	0.610
ASA				0.741
ASA 1	3 (4.2)	2 (6.2)	1 (2.5)	
ASA 2	52 (73.2)	23 (71.9)	29 (74.4)	
ASA 3	16 (22.6)	7 (21.9)	9 (23.1)	
Previous abdominal surgery	32 (45.1)	11 (34.4)	21 (53.8)	0.100
Neoadjuvant therapy	9 (12.7)	0 (0)	9 (23.1)	0.0036
Venous involvement	12 (16.9)	4 (12.5)	8 (20.5)	0.370
Vascular anomalies	18 (25.3)	9 (28.1)	9 (23.1)	0.626
Preoperative biliary drainage	36 (50.7)	14 (43.8)	22 (56.4)	0.288

Values in parenthesis are percentages unless indicated otherwise: * Numbers are expressed as median and interquartile range. MIPD: minimally invasive pancreaticoduodenectomy, LPD: laparoscopic pancreaticoduodenectomy, RPD: robotic pancreaticoduodenectomy, BMI: body mass index, ECOG PS: Eastern Cooperative Oncology Group Performance Status, ACCI: Age-adjusted Charlson Comorbidity Index, ASA: American Society of Anesthesiologists Physical Status Classification System.

**Table 2:** Intraoperative outcomes of patients undergoing MIPD.

Characteristic	Overall (71)	LPD (32)	RPD (39)	*p*
Vascular resection				0.059
No	66 (92.9)	32 (100)	34 (87.2)	
PV	1 (1.5)	0 (0)	1 (2.5)	
SMV	4 (5.6)	0 (0)	4 (10.3)	
Operative time *	545 (500–600)	555 (510–597.5)	540 (480–620)	0.561
Resection time *	310 (265–350)	305 (271.2–328.7)	318 (260–370)	0.708
Conversion	0 (0)	0 (0)	0 (0)	/
Wirsung diameter (mm) *	4 (2–5)	4 (2–5)	4 (2–5)	0.276
Blood loss (mL)				0.231
<400	49 (69.0)	25 (78.1)	24 (61.5)	
401–700	13 (18.4)	4 (12.5)	9 (23.1)	
701–1000	6 (8.4)	3 (9.4)	3 (7.7)	
>1000	3 (4.2)	0 (0)	3 (7.7)	
Blood loss (mL) *	250 (150–500)	300 (200–487.5)	200 (150–600)	0.561
Pancreatic texture				0.283
Soft	24 (33.8)	9 (28.1)	15 (38.5)	
Intermediate	7 (9.9)	5 (15.6)	2 (5.1)	
Firm	40 (56.3)	18 (56.3)	22 (56.4)	
FRS *	3 (1–5)	2 (0.2–5)	3 (1–4)	0.706
FRS moderate-high	35 (49.3)	15 (46.9)	20 (51.3)	0.711
Class of FRS				0.146
Negligible (0 pt)	15 (21.1)	8 (25.0)	7 (17.9)	0.640
Low (1–2 pt)	20 (28.2)	9 (28.1)	11 (28.2)	
Moderate (3–6 pt)	31 (43.7)	14 (43.8)	17 (43.6)	
High (7–10 pt)	5 (7.0)	1 (3.1)	4 (10.3)	

Values in parenthesis are percentages unless indicated otherwise: * Numbers are expressed as median and interquartile range. MIPD: minimally invasive pancreaticoduodenectomy, LPD: laparoscopic pancreaticoduodenectomy, RPD: robotic pancreaticoduodenectomy, PV: portal vein, SMV: superior mesenteric vein, FRS: Fistula risk score.

**Table 3:** Postoperative outcomes of patients undergoing MIPD.

Characteristic	Overall (71)	LPD (32)	RPD (39)	*p*
Length of stay (day) *	11 (8–16)	10.5 (8–19.2)	11 (8–16)	0.893
Overall complications	32 (45.1)	13 (40.6)	19 (48.7)	0.495
Reoperation	4 (5.6)	3 (9.4)	1 (2.5)	0.215
Complications:				0.551
1	40 (56.3)	20 (62.6)	20 (51.3)	
2	14 (19.7)	4 (12.5)	10 (25.7)	
3a	10 (14.1)	4 (12.5)	6 (15.5)	
3b	3 (4.2)	2 (6.2)	1 (2.5)	
4	1 (1.5)	0 (0)	1 (2.5)	
90-day mortality	3 (4.2)	2 (6.2)	1 (2.5)	0.442
Comprehensive CI *	0 (0–25.7)	0 (0–24.9)	0 (0–25.7)	0.587
Severe complications	15 (21.1)	8 (25.0)	7 (17.9)	0.468
POPF	6 (8.4)	3 (9.4)	3 (7.7)	0.799
POPF grade				0.691
Grade B	3 (4.2)	1 (3.1)	2 (5.0)	
Grade C	3 (4.2)	2 (6.2)	1 (2.5)	
DGE	11 (15.5)	5 (15.6)	6 (15.4)	0.977
DGE grade				0.330
Grade A	4 (5.6)	3 (9.4)	1 (2.5)	
Grade B	7 (9.9)	2 (6.2)	5 (12.8)	
Grade C	0 (0)	0 (0)	0 (0)	
Biliary leak	4 (5.6)	3 (9.4)	1 (2.5)	0.215
Biliary leak grade				0.280
Grade A	2 (2.8)	1 (3.1)	1 (2.5)	
Grade B	2 (2.8)	2 (6.2)	0 (0)	
Grade C	0 (0)	0 (0)	0 (0)	
PPH	7 (9.9)	3 (9.4)	4 (10.3)	0.901
PPH grade				0.669
Grade A	1 (1.5)	0 (0)	1 (2.5)	
Grade B	3 (4.2)	1 (3.1)	2 (5.0)	
Grade C	3 (4.2)	2 (6.2)	1 (2.5)	
90 day-readmission	6 (8.4)	2 (6.2)	4 (10.3)	0.545
IAFA	9 (12.7)	3 (9.4)	6 (15.4)	0.499

Values in parenthesis are percentages unless indicated otherwise: * Numbers are expressed as median and interquartile range. MIPD: minimally invasive pancreaticoduodenectomy, LPD: laparoscopic pancreaticoduodenectomy, RPD: robotic pancreaticoduodenectomy, CI: Complication Index, POPF: Postoperative Pancreatic Fistula, DGE: Delayed Gastric Emptying, PPH: Post Pancreatectomy Hemorrhage, IAFA: Ineffective Artery-First Approach.

**Table 4:** Univariate and multivariate analyses for IAFA among patients undergoing MIPD.

	Univariate Analysis		Multivariable Analysis	
	OR (95% CI)	*p* Value	OR (95% CI)	*p* Value
Sex (male)	5.181 (0.994–27.027)	0.035	5.181 (1.105–37.037)	0.036
Age > 70	1.029 (0.252–4.203)	0.968		
BMI > 30	0.982 (0.106–9.067)	0.987		
ASA > 2	3.333 (0.776–14.325)	0.092	3.310 (0.687–15.605)	0.131
ACCI > 5	1.620 (0.397–6.620)	0.498		
Previous abdominal surgery	2.245 (0.387–13.013)	0.319		
Vascular involvement	0.580 (0.066–5.120)	0.620		
RHA anomalies	0.821 (0.154–4.369)	0.817		
Preoperative biliary drainage	2.133 (0.489–9.303)	0.305		
Ca 19.9 > 500 U/mL	0.680 (0.075–6.124)	0.730		
Neoadjuvant chemotherapy	2.245 (0.387–13.013)	0.319		
Robotic approach	1.758 (0.403–7.667)	0.449		
Tumor size > 25 mm	1.620 (0.397–6.620)	0.380		
Positive lymph nodes (yes)	0.844 (0.206–3.457)	0.814		

IAFA: Ineffective Artery-First Approach, MIPD: minimally invasive pancreaticoduodenectomy, OR: odds ratio, CI: confidence interval, BMI: body mass index, ASA: American Society of Anesthesiologists Physical Status Classification System, ACCI: Age-adjusted Charlson Comorbidity Index, RHA: right hepatic artery.

**Table 5:** Pathological and oncological outcomes of patients undergoing MIPD.

Characteristic	Overall (71)	LPD (32)	RPD (39)	*p*
Tumor size (mm) *	25 (20–30)	25 (20–30)	25 (21–30)	0.847
Grading				0.281
not applicable	3 (4.2)	0 (0)	3 (7.7)	
Gx	1 (1.5)	0 (0)	1 (2.5)	
G1	0 (0)	0 (0)	0 (0)	
G2	36 (50.7)	16 (50.0)	20 (51.3)	
G3	31 (43.7)	16 (50.0)	15 (38.5)	
Perineural invasion	47 (66.2)	25 (78.1)	28 (71.8)	0.593
Lymphovascular invasion	37 (52.1)	16 (50.0)	21 (53.8)	0.746
Lymph nodes harvested *	22 (15–27)	17 (15–24.7)	24 (17–28)	0.023
Positive lymph nodes *	1 (0–3)	1 (0–3)	1 (0–3)	0.630
pN+	42 (59.1)	21 (65.7)	21 (53.8)	0.315
Lymph node ratio *	0.05 (0–0.12)	0.06 (0–0.16)	0.04 (0–0.11)	0.291
R0	53 (74.6)	26 (81.2)	27 (69.2)	0.246
R1				0.108
Posterior margin	14 (19.7)	6 (100)	8 (66.7)	
Medial margin	4 (5.6)	0 (0)	4 (33.3)	
pT				0.590
pT0 ^†^	2 (2.8)	0 (0)	2 (5.1)	
pTis	1 (1.5)	0 (0)	1 (2.5)	
pT1a	3 (4.2)	1 (3.1)	2 (5.1)	
pT1b	3 (4.2)	1 (3.1)	2 (5.1)	
pT1c	15 (21.1)	6 (18.7)	9 (23.1)	
pT2	43 (60.6)	21 (65.7)	22 (56.4)	
pT3	4 (5.6)	3 (9.4)	1 (2.5)	
pN				0.570
pN0	29 (40.9)	11 (34.4)	18 (46.2)	
pN1	29 (40.9)	15 (46.9)	14 (35.9)	
pN2	13 (18.4)	6 (18.7)	7 (17.9)	
TNM Stage > IIa	41 (57.8)	21 (65.7)	20 (51.3)	0.223
TNM Stage				0.577
Ia	16 (22.6)	5 (15.6)	11 (28.2)	
Ib	13 (18.4)	6 (18.7)	7 (17.9)	
IIa	1 (1.5)	0 (0)	1 (2.5)	
IIb	28 (39.1)	15 (46.9)	13 (33.5)	
III	13 (18.4)	6 (18.7)	7 (17.9)	
Performed adjuvant therapy	40/67 ^††^ (59.7)	20 (62.6)	20/35 ^†^ (57.1)	0.655

Values in parenthesis are percentages unless indicated otherwise: * Numbers are expressed as median and interquartile range. MIPD: minimally invasive pancreaticoduodenectomy, LPD: laparoscopic pancreaticoduodenectomy, RPD: robotic pancreaticoduodenectomy. ^†^ pT0 indicates patients undergoing neoadjuvant treatment with no evidence of residual tumor on the surgical specimen. ^††^ Patients who underwent totally neoadjuvant chemotherapy were excluded. For pTNM stage the 8th edition of AJCC TNM staging system was used.

## Data Availability

The data presented in this study are available on request from the corresponding author due to their containing information that could compromise the privacy of research participants (specify the reason for the restriction).

## Abbreviations

The following abbreviations are used in this manuscript:

PD	Pancreatoduodenectomy
SMA	Superior Mesenteric Artery
SMV	Superior Mesenteric Vein
AFA	Artery-first Approach
IPDA	Inferior Pancreatico-duodenal Artery
MIPD	Minimally Invasive Pancreaticoduodenectomy
PC	Pancreatic Cancer
LPD	Laparoscopic Pancreaticoduodenectomy
FRS	Fistula Risk Score
BMI	Body Mass Index
LN	Lymph Node
IAFA	Ineffective Artery-first Approach
